# Supplementary material for: LOng COvid Multidisciplinary consortium Optimising Treatments and servIces acrOss the NHS (LOCOMOTION): protocol for a mixed-methods study in the UK
Source: BMJ Open. 2022 May 16;12(5):e063505. doi: 10.1136/bmjopen-2022-063505 (PMC9114312; doi:10.1136/bmjopen-2022-063505)
Supplement: Reviewer comments [file bmjopen-2022-063505.reviewer_comments.pdf]

PEER REVIEW HISTORY

BMJ Open publishes all reviews undertaken for accepted manuscripts. Reviewers are asked to complete a checklist review form (**http://bmjopen.bmj.com/site/about/resources/checklist.pdf**) and are provided with free text boxes to elaborate on their assessment. These free text comments are reproduced below.

ARTICLE DETAILS

**TITLE (PROVISIONAL)**	LOng COvid Multidisciplinary consortium Optimising Treatments and servIces acrOss the NHS (LOCOMOTION): protocol for a mixed-methods study in the UK
**AUTHORS**	Sivan, Manoj; Greenhalgh, Trisha; Darbyshire, Julie; Mir, Ghazala; O'Connor, Rory J.; Dawes, Helen; Greenwood, Darren; O'Connor, Daryl; Horton, Mike; Petrou, Stavros; de Lusignan, Simon; Curcin, Vasa; Mayer, Erik; Casson, Alexander; Milne, Ruairidh; Rayner, Clare; Smith, Nikki; Parkin, Amy; Preston, Nick; Delaney, Brendan

This article was not externally reviewed at BMJ Open. Protocols that have been independently assessed prior to submission to BMJ Open are usually fast-tracked to publication on the grounds that further substantial changes will not be possible. This independent assessment will usually be external, independent review for both a substantial grant award from a non-commercial or government funder and ethics approval.
